# Multi-scale modeling for the transmission of influenza and the evaluation of interventions toward it

**DOI:** 10.1038/srep08980

**Published:** 2015-03-11

**Authors:** Dongmin Guo, King C. Li, Timothy R. Peters, Beverly M. Snively, Katherine A. Poehling, Xiaobo Zhou

**Affiliations:** 1 Department of Radiology, Wake Forest University School of Medicine, Winston-Salem, NC, USA; 2 Department of Pediatrics, Wake Forest University School of Medicine, Winston-Salem, NC, USA; 3 Department of Biostatistical Sciences, Wake Forest University School of Medicine, Winston-Salem, NC, USA; 4 Department of Epidemiology and Prevention, Wake Forest University School of Medicine, Winston-Salem, NC, USA

## Abstract

Mathematical modeling of influenza epidemic is important for analyzing the main cause of the epidemic and finding effective interventions towards it. The epidemic is a dynamic process. In this process, daily infections are caused by people's contacts, and the frequency of contacts can be mainly influenced by their cognition to the disease. The cognition is in turn influenced by daily illness attack rate, climate, and other environment factors. Few existing methods considered the dynamic process in their models. Therefore, their prediction results can hardly be explained by the mechanisms of epidemic spreading. In this paper, we developed a heterogeneous graph modeling approach (HGM) to describe the dynamic process of influenza virus transmission by taking advantage of our unique clinical data. We built social network of studied region and embedded an Agent-Based Model (ABM) in the HGM to describe the dynamic change of an epidemic. Our simulations have a good agreement with clinical data. Parameter sensitivity analysis showed that temperature influences the dynamic of epidemic significantly and system behavior analysis showed social network degree is a critical factor determining the size of an epidemic. Finally, multiple scenarios for vaccination and school closure strategies were simulated and their performance was analyzed.

Due to the annual recurrence of influenza, mathematical and computational models have been used widely in epidemiology to describe pandemic and seasonal transmission of virus and to conduct the simulations for the analysis and control toward it. One classical approach is to use statistical agent-based modeling to build likelihood functions with respect to person-to-person transmission probabilities to describe the spread of influenza globally^1^,^2^. This method often assumes individuals are homogeneous and does not account for interpersonal interactions and individuals' personalities, which are key factors influencing the transmission of influenza virus. Another classical approach is using stochastic network model by incorporating interactions based on geography, demography and migration information to discover transmission patterns within heterogeneous populations^3^,^4^,^5^,^6^. Those methods mentioned above studied the transmission among various regions, for example, within communities^7^,^8^, cities^9^, countries^2^,^10^, or worldwide^3^,^4^,^6^. The purposes of these studies are to evaluate specific intervention strategies, such as school closure^11^, vaccination^1^,^12^,^13^, isolation of cities^12^, and to estimate the impact of human mobility locally and globally^14^,^15^,^16^.

An epidemic is a dynamic process. In this process, daily infections are caused by people's contacts, and the frequency of contacts can be mainly influenced by their cognition to the disease. The cognition is in turn influenced by daily illness attack rate reported by media broadcasts^17^,^18^, climate^19^, and other environment factors. The association between human cognitive behavior and virus transmission has been investigated through cognitive behavioral theories^20^,^21^,^22^, showing highly daily virus attack rate evokes people's precaution to the disease. They may adopt protective behaviors in order to avoid being infected, resulting in the reduction of the daily virus attack rate. However, few existing methods considered the dynamic process in their models. Therefore, their prediction results can hardly be explained by the mechanisms of epidemic spreading. In this paper, we develop a heterogeneous graph modeling approach (HGM) to describe the dynamic process of influenza virus transmission in order to increase the validity, interpretability, and utility of network modeling by taking advantage of our unique clinical data.

We have enrolled 4870 patients including both clinical information and demographic information in two hospitals of Forsyth County, North Carolina, USA serving more than 94% of residents in this area for four consecutive influenza seasons of 2009–2010 through 2012–2013^23^. We also used the 2005–2009 U.S. Synthetic Population Database from RTI International to build social network of this county^24^. The population information we used includes each household's geographical location and family members, each family member's workplace and school, each individual's demographic and socioeconomic information and locations for their activities, as well as the assignments of students to schools and assignments of workers to workplaces. We then embedded an Agent-Based Model (ABM) in the HGM to describe the dynamic change of an epidemic influenced by individuals' cognition and reaction to the epidemic, climate, and degree of social network. The most important is, our HGM approach was trained and validated by clinical data. This approach can be used to create the transmission of influenza *in silico*, analyze the agent behavior under conditions of influenza spread, and simulate the therapeutic and non-therapeutic interventions to prevent or mitigate it.

## Results

We simulated the epidemic spread in Forsyth Country, NC, USA (see Methods). Simulations of local epidemic spread are conducted using our heterogeneous graph model (HGM) approach. Generally, the HGM consists of two layers. The first layer describes the social structure of studied region using open-access synthetized populations that capture the demographic and geographic heterogeneities of population in this region. The second layer is an ABM that embedded in the HGM to describe how the virus is transmitted through individuals' interaction and social behavior, taking into account the influence brought by individuals' cognition and reaction to the epidemic, climate, degree of social network, and other environmental factors. In the model, individuals were assigned to child care centers, schools, workplaces, and public areas based on their ages. A day is divided into Outside Time and Family Time. In outside time, people's contacts occur at child care centers, schools, workplaces, and public areas. Family time is from the moment people enter home to the moment they leave home in the next morning. We simply assume that during this period, they only have chance to contact with their family members. The interactions between people who go into public areas after work hours are considered as activities in Outside Time as well. We assume a certain proportion of individuals go to public areas after work hours and the number is influenced by daily infection rate. In severe epidemic, individual tends to avoid staying in public area in order to protect themselves. According to our clinical data, the simulated epidemics were from the 40^th^ week of the year (beginning of October) to the 13^th^ week of the next year (end of March). Public holidays and schools' winter break were involved in the simulation as well. Individuals' activities and social behaviors during holidays were set the same as on weekends. Children at schools had winter break during the 52^nd^ week of the year and half of the 1^st^ week of the next year. During this period, their activities were also set the same as on weekends.

### Simulation of epidemics

**Figure 1:**
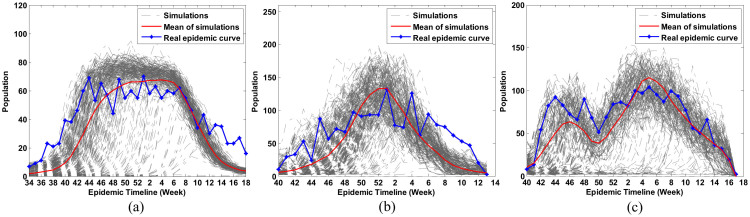
Simulation of epidemics. Simulated influenza clinical cases (red line) were obtained from the average of 300 simulations (gray line). Processed real epidemic (blue line) is the actual epidemic timeline, from data collected from hospitals in Forsyth Country. (a) 2009–2010 influenza season, (b) 2010–2011 influenza season, and (c) 2011–2012 influenza season.

The epidemics of influenza in 2009–2010, 2010–2011, and 2011–2012 were simulated separately. Blue lines in Figure 1 show the number of patients who went to hospital in the studied area weekly. They had very different patterns in the three influenza seasons. In the 2009–2010 influenza season, the number of infected individuals rose rapidly within four weeks, from the 40^th^ week to the 44^th^ week of 2009. It then remained almost stable for 15 weeks, from the 44^th^ week of 2009 to the 7^th^ week of 2010. In the 2010–2011 influenza season, the number of infected individuals rose gradually and reached a maximum in the first week of 2011, and dropped slowly until the end of the influenza season. Different from the two epidemics, however, the epidemic of 2011–2012 influenza season had two waves. Red lines in Figure 1 are the predicted epidemic dynamics of the three influenza seasons, obtained from the average of 300 simulations (gray lines). Compared to the existing network modeling methods^6^,^9^,^15^,^25^ (see Supplementary Fig. S3–S4 online), our HGM approach can create a daily feedback of human behavior to the epidemic by including Equations (1) and (2) in the ABM and trained by clinical data (see Methods). Therefore it can simulate the dynamic change of an epidemic and produce distinct epidemic patterns.

Individuals' cognitive behavior toward an epidemic can impact their interaction and virus transmission, resulting in dynamic changes of the epidemic. This feedback process has been included in our ABM. To measure the function of individual's cognition and caution against the disease, we conducted experiments by setting *H*(*i*,*t*) and *J*(*i*,*t*) (see Methods) as constant throughout the influenza season (see Supplementary Fig. S5 online). The results showed that HGM with constant cognition functions could not represent the dynamic change of the epidemic and failed to model the epidemics with multi-waves, because no cognitive and protective behavior was evoked to prevent the increasing of infections.

In the simulation of 2010–2011 influenza epidemic, the basic reproductive number R0, the average number of individuals a typical infected person infects during his or her infectious period, was 1.26. The average connections per person and illness attack rate with respect to different places and age groups were listed in Table 1. The entire results of the simulations of the three influenza seasons can be found as Supplementary Table S3 online. All simulations showed children were the most vulnerable group. The younger a child was, the more possibly he or she got infected. Children have more connections than adults; this high connectivity resulted in a high probability of virus transmission and illness attack rate. This finding was proved by our clinical data. In the studied county, the percentage of persons under 18 years is 23.8%^26^, while clinical data showed that 34.8% of influenza patients were children under 18. Once they are infected, they will spread the virus to family members and people around them. Therefore, extent and severity of epidemic influenza largely depends on virus transmission in children^27^,^28^. Specific interventions toward children, such as vaccination and school closure, have been advocated to prevent an epidemic^29^,^30^,^31^,^32^,^33^. In this paper, we will simulate multiple scenarios for vaccination and school closure strategies and evaluate their performance to provide suggestions about the adoption of the interventions.

### Sensitivity analysis

We performed parameter sensitivity analysis to examine the robustness of our model and to evaluate whether varying key parameters affected the results. As shown in Equation (3) (see Methods), *r*(*i*,*t*), the risk of an individual *i* being infected at time *t* is related to daily illness attack rate *R*(*t*), local temperature *T*(*t*), his or her social network degree *D*(*i*,*t*), and the age *A*(*i*). Parameters *c_r_*, *c_t_*, *c_d_* and *c_a_* are the coefficients of function *f*(*R*(*t*)), *f*(*T*(*t*)), *f*(*D*(*i*,*t*)), and *f*(*A*(*i*)), respectively. *b_r_*, *b_t_* and *b_d_* are corresponding deviations. We varied these key parameters to see if their changes can strongly impact the total infection number.

**Figure 2:**
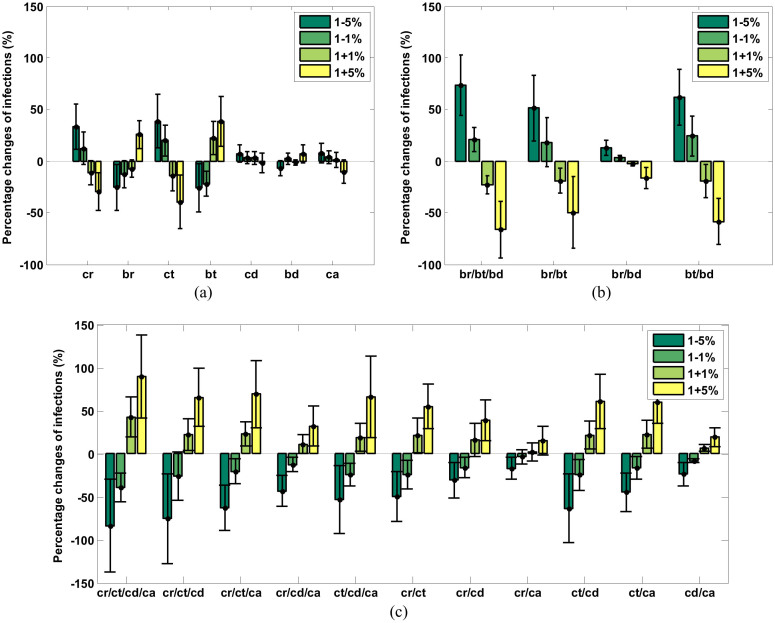
Results of the parameter sensitivity analysis of the model. (a) Single parameter sensitivity analysis, (b) multi-parameter sensitivity analysis based on parameters related to deviations, (c) multi-parameter sensitivity analysis based on parameters related coefficients. The bars show the percentage changes of total numbers of infected people when varying parameters. Each bar represents the mean percentage changes of infected individuals calculated from 300 simulations. The error bars represent the 95% confidence interval (CI) of the means.

Figure 2 is the bar chart showing the percentage changes of infected individuals when varying parameters with respect to the values using default parameters listed in Table S2 for the simulation of 2010–2011 influenza season. Each bar represents the mean percentage changes of infected individuals calculated from 300 simulations. The error bars represent the 95% confidence interval (CI) of the means. In Figure 2(a), the sensitivity analysis was done on single parameter. Figure 2(b) shows the results of multi-parameter sensitivity analyses based on deviations in Equations (1) and (2). Figure 2(c) shows the results of multi-parameter sensitivity analyses based on coefficients in Equations (1) and (2). We did not mix deviations and coefficients together to make multi-parameter sensitivity analyses because coefficients are positively correlated with number of infections, while deviations have reverse correlation with them. In the three figures, the tick labels along the x-axis indicate which parameter (Figure 2(a)) or which groups of parameters (Figure 2(b) and Figure 2(c)) are varied. We only varied one parameter or one group of parameters by reducing and increasing their values 1% and 5% from their default values, with the rest of parameters fixed.

Figure 2(a) shows that, the most sensitive variables are *c_r_*, *b_r_*, *c_t_* and *b_t_*, which are related to illness attack rate and local temperature. Figure 2(b) and Figure 2(c) show that, if a group includes parameters related to local temperature (*c_t_* or *b_t_*), varying the parameters in the group results in bigger change of the infection number. Hence, changes in illness attack rate and climate, especially temperature, impact the epidemic more than other factors. In fact, links between climate and influenza virus transmission have been identified^19^,^34^. Cooling of the nasal mucosa is thought to increase the viscosity of the mucous layer and reduce the frequency of cilia beats^34^. In this way, breathing cold air would slow mucociliary clearance and thereby encourage viral spread within the respiratory tract^19^. Additionally, in cold weather, the duration of peak viral shedding is longer than warm weather^19^. The increased shedding may lead to the increased transmission.

### System behavior analysis

Two inputs of the system, initial infection number at the beginning of simulation and number of weekly exotic infections may influence the output of the system. Initial infection number is the number of infected individuals on the first day of simulated influenza season. We assigned the default value of initial infection number to 0.01% of total simulated population by referring the record of our clinical data. Number of weekly exotic infections is the total number of infected individuals that come into the studied area by air traffic in a week. We assigned the default value of weekly exotic infections to 0.04% of total simulated population based on our investigation on the passenger capacity of the airport around the simulated area.

We perturbed the two input variables to see whether the output of the system can reach stable status eventually. The perturbation was under three conditions, with the degree of 6.7, 8.7, and 10.7, respectively. Network degree is defined as the average connections between individuals. 6.7 was its default value in our simulation, computed through the optimized system under the supervision of clinical data (see Methods). We varied both of the input variables from 0.01% to 0.12% of total simulated population. In each simulation, we only perturbed one variable and fixed the other at its default value.

**Figure 3:**
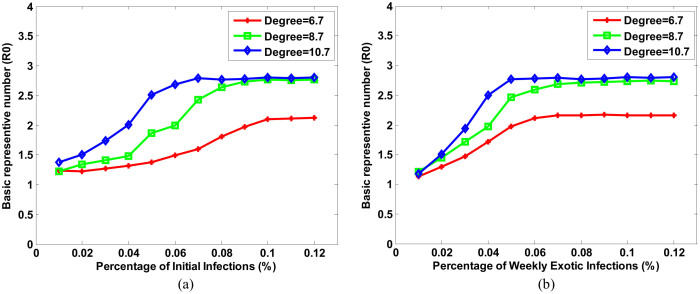
System behavior analyses by perturbing two critical variables with specific social network degree. (a) Initial infection, (b) weekly exotic infections.

Figure 3 illustrates the analysis results. The output of the system was characterized by R_0_. Figure 3(a) shows the change of R_0_ with respect to the initial infection numbers. The relationship between initial infections and R_0_ approximately follows a sigmoid function (Figure 3). In general, R_0_ rose with the increase of initial infection numbers until a certain initial infection numbers has reached. We call the rate of “certain initial infection number” over total simulated population as a “**State Point**”. The red curve in Figure 3(a) represents the case with the degree of social network being 6.7. The simulation shows R_0_ converges to 2.12 when the **State Point** is 0.1%. Similarly, the green curve represents the case with the degree of social network being 8.7, showing R_0_ converges to 2.76 when the **State Point** is 0.08%. And the blue curve shows R_0_ converges to 2.79 when the **State Point** is 0.05% with the degree of social network being 10.7.

Figure 3(b) is the change of R_0_ with respect to weekly exotic infections, showing that R_0_ was eventually stable with the increasing of weekly exotic infections. Same as Figure 3(b), in this figure, red, green, and blue curve represents the case with the degree of social network being 6.7, 8.7, and 10.7, respectively. In the first case (degree = 6.7), R_0_ converges to 2.16 when the **State Point** is 0.07%. In the second case (degree = 8.7), R_0_ converges to 2.73 when the **State Point** is 0.08%. And in the last case (degree = 10.7), R_0_ converges to 2.79 when the **State Point** is 0.05%.

In summary, both of the figures showed that, (1) R_0_ reaches a maximum at **State Point** and trends to a stable status. During an epidemic, once infected individual is recovered, he or she will be immune to subsequent infections. When more and more individuals are immune, viruses have low chance to spread. Therefore, the number of infections can hardly increase, and the accumulation of total infections tends to be a fixed number. (2) R_0_ is positively correlated to social network degree. In the region with higher social network degree, individuals have more chance to contact with each other, increasing the possibility of virus transmission between individuals. It will lead to a larger size of epidemic. Therefore, severe epidemic often happens in the region where individuals have more connections, because viruses are transmitted much easier and faster in the region. (3) Different degree leads to different **State Point**. Generally, smaller social network degree results in bigger **State Point**. It means in the region individuals have fewer connections, influenza viruses have low chance to spread. Therefore, severe epidemic does not frequently and easily happen unless initial infection number or exotic infection number is extremely large. Consequently, social network degree is a critical factor determining the magnitude of an epidemic. The reduction of network degree is important in the control of epidemic. In fact, many interventions against the epidemic are related to the decrease of network degree, such as school closure and patient self-isolation.

### Simulation of interventions

**Figure 4:**
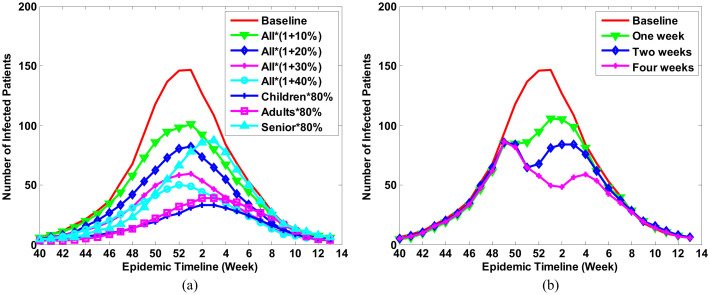
Simulation of the influenza epidemic curve with different interventions. (a) Vaccination. Seven scenarios were simulated for this intervention. In the first four scenarios, the coverage of vaccination is enlarged by 10%, 20%, 30%, and 40% in all population, respectively. In the last three scenarios, the coverage of vaccination was set as 80% in children, adults, and seniors, respectively; and (b) School closure. Three scenarios were simulated in which the length of school closure was one week, two weeks, and four weeks. The time of school closure started when more than 0.1% of populations go to a hospital due to influenza infection. Baseline means there was no intervention and R_0_ = 1.26.

Vaccination and school closure are two common interventions in an epidemic. However, which one is more suitable to a specific epidemic and when a community should adopt these interventions is hard to predict. Figure 4 presents the simulations of an influenza epidemic curve under various interventions. All intervention scenarios were simulated under the same magnitude of epidemic (baseline, R_0_ = 1.26).

Figure 4(a) shows the predicted epidemic curves given different coverage of vaccination. National reports from the Centers for Disease Control and Prevention (CDC) of the United States showed that from 2009 to 2012, the average influenza vaccine coverage was 46.27%, 35.53%, and 67.07% for persons 0.5–17 years, 18–64 years, and 65+ years of age, respectively^35^. It indicates the vaccination coverage still can be increased. We therefore simulated the scenarios when the vaccination coverage is increased by 10%, 20%, 30%, and 40% in the whole population. Additionally, research has shown that in clinically-confirmed cases, influenza vaccine effectiveness (VE) in persons under 4 years of age, 5–17, 18–64, and 65+ was 63%, 56%, 51%, and 36%, respectively^36^. VE differs substantially in different age groups. Here we modeled the efficiency of intervention when only a specific age group is 80% vaccinated. We set the maximal percentage of the vaccinated population as 80% because some individuals cannot be vaccinated due to a health condition. The simulations suggested vaccinating only toward children can prevent the outbreak. This finding is reinforced by Table 1, showing that the illness attack rate among children under 4 years old is 59.60% and among children with 5–17 years old is 14.98%. The numbers are far bigger than those among adults and older adults. Thus, immunizing children and adolescents with influenza vaccine could significantly protect them from influenza virus and prevents their infecting family members^29^,^30^.

Figure 4(b) presents the simulation of school closure. The interventions started when more than 0.1% of the population goes to a hospital due to influenza infection since the beginning of the influenza season. Generally, the rate of infection is bigger than 0.1% because a large number of infected patients choose stay at home and use nonprescription medicines. It was reported that closing school was very effective within the first 30 days and the infection rate was hardly to be reduced if the closing lasts more than 30 days^37^. Besides, school closure brings high economic cost^33^. Here we only simulated this intervention within four weeks. Three scenarios were simulated in which the length of school closure was one week, two weeks, and four weeks. In any scenario, when schools were reopened, the number of infected patients increased again and the outbreak recurred eventually. The simulation results showed that closing school for two weeks can only reduce the R0 from 1.26 to 0.95. School closure may not be an appropriate intervention in moderate epidemic. We can choose other low-cost interventions since the epidemic is controllable.

From Figure 4(b) we have an interesting finding. We have mentioned that holidays were already considered in our simulation. Individuals' activities and social behaviors during holidays were set the same as on weekends. Red line in Figure 4(b) is the simulated epidemic under this condition. The other three lines in Figure 4(b) represented the simulated epidemic when conducted school closure interventions. Interestingly, with school closure intervention the weekly infections dropped extremely, but during holidays and winter breaks there is no obvious decline of weekly infections. From these results we reached different conclusion from some existing claims that holidays lead to a reduction of influenza transmission^11^. Our conclusion is holiday cannot help reduce virus transmission if individuals' activities and social behaviors during holidays are the same as on weekends. It is because individuals have different recognition of holiday and school closure. During holiday, people do not need intentionally avoid crowded places. On the contrary, they are more likely to travel around, which might aggravate the epidemic. School closure often happens when the epidemic is server. People will consciously avoid place where many people gather. There is a tight association between human cognitive behavior and virus transmission. That is why school closure and holiday have essential difference in the control of disease spread and holiday cannot replace the role of school closure.

**Figure 5:**
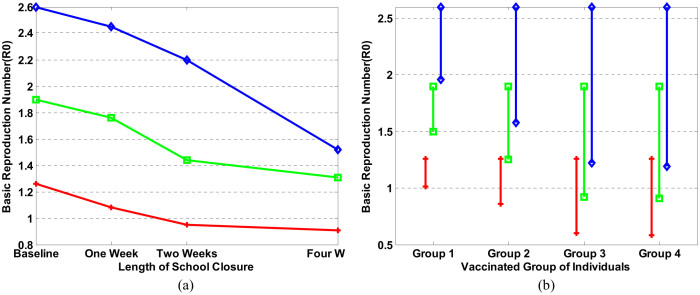
Comparison of interventions in response to three different sizes of epidemic. The baseline is R_0_ = 1.26. Another two severe epidemics (R_0_ = 1.9 and R_0_ = 2.6) were simulated as well. Red lines indicate the case of R_0_ = 1.26; green lines show intervention results when R_0_ = 1.9, and blue lines present the case when R_0_ = 2.6. (a) Vaccination. (b) School closure.

We also simulated two other severe epidemics (R_0_ = 1.9 and R_0_ = 2.6) by increasing the social network degree and exotic infections that come into the studied area by air traffic. Figure 5 presents the comparison of interventions in response to moderate and severe epidemics. In Figure 5(a), we modeled the efficiency of vaccination adopted in four targeted groups under the three epidemics. In Group 1, we assumed 80% of children in child care centers and schools were vaccinated; in Group 2, we assumed 80% of children were vaccinated; in Group 3, we assumed 80% of children and 80% of people in the workplace were vaccinated; and in Group 4, we assumed 80% of the whole population was vaccinated. The baseline was the officially reported coverage (46.27%, 35.53%, and 67.07% for persons 0.5–17 years, 18–64 years, and 65+ years of age, respectively). In a severe epidemic (R_0_ = 1.9 and R_0_ = 2.6), interventions on Group 1 helped reduce R_0_ from 2.6 to 1.96, or from 1.9 to 1.50. In both cases, the epidemics were still out of control. Such a strategy is not enough in a severe epidemic. If we enlarged the vaccination range to all children (Group 2), in the very severe epidemic (R_0_ = 2.6), R_0_ was reduced to 1.58 and the epidemic was still out of control, but this strategy prevented the epidemics of both R_0_ = 1.9 and 1.26 very well. These results suggested that vaccination is more suitable when the epidemic is not very severe. In Groups 3 and 4, 80% vaccination resulted in a sharp decrease of R_0_ in each simulated epidemic, while R_0_ had almost the same reduction pattern in both groups. These results indicated that a vaccination strategy targeting children and people in the workplace can mitigate an epidemic; urging mass vaccination of a whole population is not necessary.

Figure 5(b) shows the impact of school closure duration on R_0_. Baseline is no school closure at all. In any case, school closure starts when more than 0.1% population goes to the hospital due to influenza infection. The duration of school closure is one week, two weeks, and four weeks, respectively. In all of the three epidemics, all values of R_0_ dropped even though school was only closed for one week. However, if the epidemic was moderate, for instance, R_0_ = 1.26, the value of R_0_ did not reduce appreciably if schools were kept closed for more than 2 weeks. On the other hand, closing schools for four weeks helped reduce R_0_ from 2.6 to 1.52 in a very severe epidemic. School closure was especially useful in a severe epidemic^31^,^32^. However, since it is a temporary strategy, adopting only school closure is not enough to completely prevent a severe epidemic. Combining targeted group vaccination and school closure may achieve the best result^33^.

## Discussion

In an influenza season, many factors work together leading to an influenza epidemic. These factors may include individuals' awareness of the disease (i.e. they might have strong reactions to media reports or to observing infection among their contacts), activity (i.e. people coming from infected areas may bring virus to their contacts), social behavior (i.e. higher contact frequency makes transmission faster), discrepancies between the vaccine strain and the circulating strain of influenza virus leading to decreased vaccine effectiveness, and climate (cool weather enhances the transmission of virus)^19^. These factors play an important role in the influenza virus transmission. Understanding the influences of these factors on the spread of influenza can be critical to improving the control strategies. While the studies about human's cognitive and behavioral responses to an epidemic have been reported for several years, there has been relatively little systematic investigation into their function and role in the spread of the disease^20^. In this paper, we systematically studied the influence of human cognition and behaviors to an epidemic by building a mathematical framework for incorporating above factors into an agent-based epidemic model. We built the relationship between the simulated epidemics and individuals' cognition toward an epidemic by embedding the ABM into a HGM and simulating the epidemics in three influenza season.

In order to illustrate the importance of the individual's cognitive behavior to an epidemic, we conducted experiments to systematically compare our proposed HGM with the existing epidemiological models. The results showed that, these methods only can simulate epidemic with one wave, but failed to simulate multiple waves. The main reason is, their mathematical models did not describe the feedback of the epidemic. In our model, however, agents' perceptions were influenced by daily virus attack rate. They may adopt protective behaviors in order to avoid being infected, resulting in the fluctuation of epidemic curve.

The simulation results showed that the major virus transmission happens in public places, especially child care centers and schools. Children are more likely to be infected and spread virus to their family members and people around them, because both their infectivity and duration of infection are stronger and longer than those in adults^38^.

The sensitivity analysis of our model showed that the number of infections were very sensitive to local temperature. This result is coincident with the conclusion that virus transmission occurred with greater frequency in cold temperatures^19^,^34^.

Through system behavior analysis, we found that social network degree is a critical factor determining the magnitude of an epidemic. In the region with higher population density, individuals have more chance to contact with each other, increasing the possibility of virus transmission. Therefore, the interventions related to the decrease of social network degree, such as school closure and patient self-isolation are critical to prevent the epidemic.

Our simulations suggested that vaccination is the best method in a moderate epidemic. It brings little economic and social cost compared with school closure. Current VE is not very high and still can be improved, especially in older adults. As a result, we proposed a targeted vaccination strategy that only includes children and people in workplace. School closure can significantly moderate the stress on the health care system when the epidemic is severe. As a temporary and isolated intervention, however, its action is very limited and is not necessary when the epidemic is moderate. However, when the epidemic is severe, school closure is more effective than vaccination. It can rapidly reduce the daily infection cases. The effectiveness and impact of this strategy depends on the epidemiological characteristics^31^,^32^. Combined multiple strategies may obtain the best result.

Finding the best way to prevent an epidemic is a very challenging task because interactions between individuals are highly connected and complicated in today's world. Network modeling can be a flexible tool to build a plausible world, to re-enact past epidemics and simulate future outbreaks, and to analyze the major causes leading to spread of the disease. In our future work, we will improve the HGM to increase the reliability and accuracy of prediction by gathering data on contact patterns of typical places in schools and workplace to draw a general law for the reproducing of contact patterns among the county and collecting survey data about people's perception and cognition toward an epidemic to improve our agent-based model. It will help us systematically understand the virus transmission between individuals.

## Methods

### Heterogeneous graph model (HGM)

We used a stochastic network modeling method to construct the social structure in a specific region. The population information includes synthesized households, schools, workplaces, and the assignments of individuals to these places. We constructed sub-networks 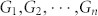 according to the population information (see Supplementary Fig. S1 online). Each of them stands for a public place, such as school and workplace. The degree of each sub-network *G_i_* is determined by two parameters: the probability that a given individual is connected to another in the same group in *p_in_* and the probability that a given individual is connected to another from a different group *p_out_*. The parameters were estimated using the Markov Chain Monte Carlo (MCMC) Metropolis-Hastings algorithm^39^ to find the best fitting of the clinical data. The parameters can be found as Supplementary Table S1 online. The whole social network was given by a block diagonal matrix *G* with *G_i_* as diagonal elements.

### Agent-based model (ABM)

We embedded an ABM in the heterogeneous graph model to describe how the virus is transmitted through individuals' interaction, taking into account the influence brought by individuals' cognition and reaction to the epidemic (see Supplementary Fig. S2 online). ABM models epidemic as a dynamic process, which is characterized by several factors such as individual's cognition, climate, degree of social network, and daily infection rate.

To model this process, we defined two functions to describe an individual's cognition to the epidemic
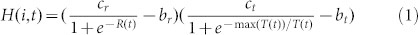
and
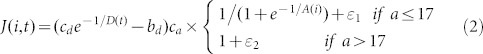
where *H*(*i*,*t*) represents the cognition of individual *i* on day *t* due to external warning, which is related to daily illness attack rate *R*(*t*) reported by media broadcasts^17^,^18^ and local temperature *T*(*t*)^19^. *J*(*i*,*t*) stands for the cognition of individual *i* on day *t* due to self-awareness, which is related to his or her social network degree *D*(*i*,*t*) and the age *A*(*i*). With the cognition, he or she may adopt protective behavior to avoiding contacting with infected individuals. Parameters *c_r_*, *c_t_*, *c_d_* and *c_a_* are constants of proportionalities, *b_r_*, *b_t_* and *b_d_* are constants of deviation. 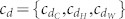 is set as three different values depending on the place the individual stays. 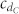 is used in the case where simulated activity happens in public places, such as the schools and the workplaces, 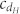 is used when simulated activity happens at home, and 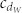 is used when simulated activity happens on weekends. *ε*_1_ and *ε*_2_ are slack variables. These parameters were estimated using the MCMC Metropolis-Hastings algorithm^39^ under the supervision of the clinical data. The parameters can be found as Supplementary Table S2 online.

Given *H*(*i*,*t*) and *J*(*i*,*t*), we defined the subject's risk of being infected on day *t*:
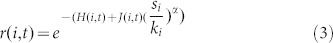
where *k_i_* indicates how many connections the individual *i* has and *s_i_* represents the amount of his or her infected connections. *α* represents the use of special prophylaxis. *α* < 1 means special prophylaxis has been used and *α* = 1 indicates there is no prophylaxis.

Finally, we obtained the force of infection of susceptible individual *i* caused by his or her infected neighbors *j* (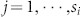) on day *t*^17^
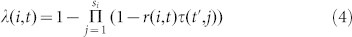
where *τ*(*t*′, *j*) is the infectivity of the infected contact *j*. *t*′ is the infection period. Infectivity *τ*(*t*′, *j*) indicates the ability of infected individual *j* to infect others. It varies with infection time *t*′ and is proportional to the amount of viral shedding in infected individuals^8^.

Infectivity of children is different from that in adults and aged individuals^40^. For the latter group, we obtained infectivity risk based on research data where viral shedding was measured in volunteers challenged with wild-type influenza viruses^41^,^42^,^43^,^44^,^45^,^46^,^47^,^48^. We mapped the viral shedding to infectivity^8^ and rescaled the infectivity curve according to the length of infectivity period. For children, we obtained their infectivity curve and the length of infection from previous work^38^,^40^,^49^. Studies confirmed that for influenza, the infectious period in adults is 5–8 days, and in children under 10 years old 7–15 days^40^,^50^. For children between 10 to 17 years old, we assumed the viral load and duration had a linear relationship with their age. We therefore rescaled the mean viral load curve to match the duration of viral shedding. Then we used the equation: *τ*(*t*′) = 0.02*V*(*t*′) to calculate the corresponding infectivity (*τ*(*t*′)) of children and adults^8^, where *V*(*t*′) is the viral load during infection. To calculate the distribution of duration of viral shedding in infectious patients, we inferred the percentage of patients who still have detectable virus in infectious period from the studies in Refs. 40, 51.

### Determine Daily Infection

People who are infected with the influenza virus typically undergo four states: *Susceptible, Exposed, Infectious*, and *Recovered* (SEIR)^52^. Individuals in each SEIR state were coded as 0, 1, 2, or 3, respectively. A state matrix *S_T_*_ × *N*_ stores each individual's SEIR state every day, where *N* is the amount of simulated individuals and *M* is number of days across the whole influenza season. Vaccinated individuals have a probability of moving a susceptible (S) individual into the recovered (R) state. We assume a portion of individuals has already been vaccinated. Influenza vaccine effectiveness^36^ and vaccination coverage^35^ are used estimate the probability of an individual's immunity. We assume all vaccinations are completed before the influenza season. In influenza season, we regarded all individuals as being in a susceptible state (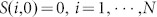). When they have contact with infectious patients on day *t*(*t* ∈ [1,*M*]), their state becomes *Exposed* (*S*(*i*,*t*) = 1). Through the Equation (4), we obtained *λ*(*i*,*t*), the force of infection of a susceptible individual *i* on day *t*(*t* ∈ [1,*M*]).

After contacting with an infected patient, the individual may or may not be infected, depending on the ability of his or her immune system. Mucosal immunity is the first line of body defense blocking influenza virus from infection, which differs by age^53^,^54^. When individuals have contact with infected patients, the strength of their mucosal immunity is used to determine the probability they will be infected. The protective status of the mucosal immune system is measured by IgA antibodies, which are secreted by mucosal surfaces and found in saliva^55^. Secretion rates of IgA indicate the level of protection offered by IgA in efficiently coating the mucosa. Research showed that the mean secretion rates of IgA in children, adults 18–64 years, and adults 65 years and older decrease with increasing age with secretion rates (Sr) of Sr = 3.84, 3.62, and 3.25 (log *μg*/2 min), respectively^54^. We therefore used the normalized secretion rate of IgA to determine the strength of an individual's mucosal immunity. We defined *mir*(*i*) as the index of mucosal immunity for each person according to his or her ages and use the following equation to determine whether the individual is infected or not.
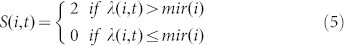
A discrete time step (half a day) was set to count the number of individuals in the four states. Every morning, before people went out, their states were recorded and updated. Every night, before they went home, their states were updated as well. *x_t_*(*t* = 1:*M*) stores a daily new infected number.

### Learning model parameters

The case study we used is in Forsyth County, North Carolina, USA because here we have performed prospective surveillance in the 2009/2010 to 2011/2012 influenza seasons in the two emergency rooms serving more than 94% of all county residents^56^. Clinical data of the three influenza seasons was used to learn parameters of our model.

We define the observation *d_obs_* using our clinical data and define **m** as a set of parameters to be estimated. The posterior probability of the parameters given by observation is defined by:
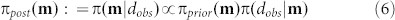
where *π_prior_* (**m**) is given by 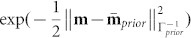 and *π*(*d_obs_* | **m**) is given by 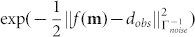. 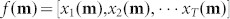 is the predicted epidemic curve. We wish to find 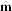 by maximizing the posterior density:
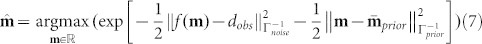
This process can be solved by a Markov Chain Monte Carlo (MCMC) Metropolis-Hastings algorithm, using the MATLAB package provided by Ref. 39.

The training process has three steps:

Step 0 (initialization): Assign all *p_in_* of the sub-network *G_i_* to the same value, and assign all *p_out_* of the sub-network *G_i_* to the same value.

Step 1 (learn network **G**), set 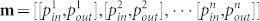, *n* is the number of sub-networks in total and fix the ABM.

Step 2 (learn ABM), fix network **G** and set **m** = [*c_r_*, *c_t_*, *c_dC_*, *c_dH_*, *c_dW_*, *b_r_*, *b_t_*, *b_d_*].

We repeat step 1 and step 2 until the biases between the simulated and real epidemic curve cannot be reduced any more. Finally, the learned parameters (see Supplementary Table S1 and S2) are used to simulate the epidemics, analyzing the factors impacting the spread of influenza, and evaluate various interventions toward it.

## Author Contributions

Conceived and designed the models: D.G., K.L. and X.Z. Performed the simulations: D.G. Analyzed the data: D.G. Contributed reagents/materials/analysis tools: K.P. and X.Z. Wrote the paper: D.G. Provided ideas to improve the system modeling: K.L., B.S., K.P. and T.P. Provided ideas about how to simulate the intervention strategies: D.G. and K.P.

## Supplementary Material

Supplementary Information Supplementary Information

## Figures and Tables

**Table 1:** Average connections per person and illness attack rate in five key places and in four age groups in the 2010-2011 influenza season

	Average connections per person	Illness attack rate
Child care center	11.26	79.39%
School	16.11	14.98%
Workplace	5.01	9.98%
Public area on workday	5.62	8.83%
Public area on weekend	8.01	11.54%
Children (0–4)	-	59.58%
Children (5–17)	-	14.97%
Adults (18–64)	-	9.47%
Seniors (65+)	-	11.26%
