# Supplementary material for: Multi-scale modeling for the transmission of influenza and the evaluation of interventions toward it
Source: Sci Rep. 2015 Mar 11;5:8980. doi: 10.1038/srep08980 (PMC4355742; doi:10.1038/srep08980)
Supplement: Supplementary Information [file srep08980-s1.pdf]

MULTI-SCALE MODELING FOR THE TRANSMISSION OF INFLUENZA AND THE EVALUATION OF INTERVENTIONS TOWARD IT

Dongmin Guo[1], King C. Li[1], Timothy R. Peters[2], Beverly M. Snively[3], Katherine A. Poehling[4*], and Xiaobo Zhou[1*]

[1]Department of Radiology, [2]Department of Pediatrics, [3]Department of Biostatistical Sciences, [4]Department of Epidemiology and Prevention, Wake Forest University School of Medicine, Winston-Salem, NC, USA.

Corresponding authors: Xiaobo Zhou (xizhou@wakehealth.edu) and Katherine A. Poehling (kpoehlin@wakehealth.edu)

Supplementary Information

Figure Legends in Paper

Figure 1. Simulation of epidemics. Simulated influenza clinical cases (red line) were obtained from the average of 300 simulations (gray line). Processed real epidemic (blue line) is the actual epidemic timeline, from data collected from hospitals in Forsyth Country. (a) 2009-2010 influenza season, (b) 2010-2011 influenza season, and (c) 2011-2012 influenza season.

Figure 2. Results of the parameter sensitivity analysis of the model. (a) Single parameter sensitivity analysis, (b) multi-parameter sensitivity analysis based on parameters related to deviations, (c) multi-parameter sensitivity analysis based on parameters related coefficients. The bars show the percentage changes of total numbers of infected people when varying parameters. Each bar represents the mean percentage changes of infected individuals calculated from 300 simulations. The error bars represent the 95% confidence interval (CI) of the means.

Figure 3. System behavior analyses by perturbing two critical variables with specific social network degree. (a) Initial infection, (b) weekly exotic infections.

Figure 4. Simulation of the influenza epidemic curve with different interventions. (a) Vaccination. Seven scenarios were simulated for this intervention. In the first four scenarios, the coverage of vaccination is enlarged by 10%, 20%, 30%, and 40% in all population, respectively. In the last three scenarios, the coverage of vaccination was set as 80% in children, adults, and seniors, respectively; and (b) School closure. Three scenarios were simulated in which the length of school closure was one week, two weeks, and four weeks. The time of school closure started

when more than 0.1% of populations go to a hospital due to influenza infection. Baseline means there was no intervention and R_0=1.26.

Figure 5. Comparison of interventions in response to three different sizes of epidemic. The baseline is R_0=1.26. Another two severe epidemics (R_0=1.9 and 2.6) were simulated as well. Red lines indicate the case of R_0=1.26; green lines show intervention results when R_0=1.9, and blue lines present the case when R_0=2.6. (a) Vaccination. (b) School closure.

Supplementary Figures

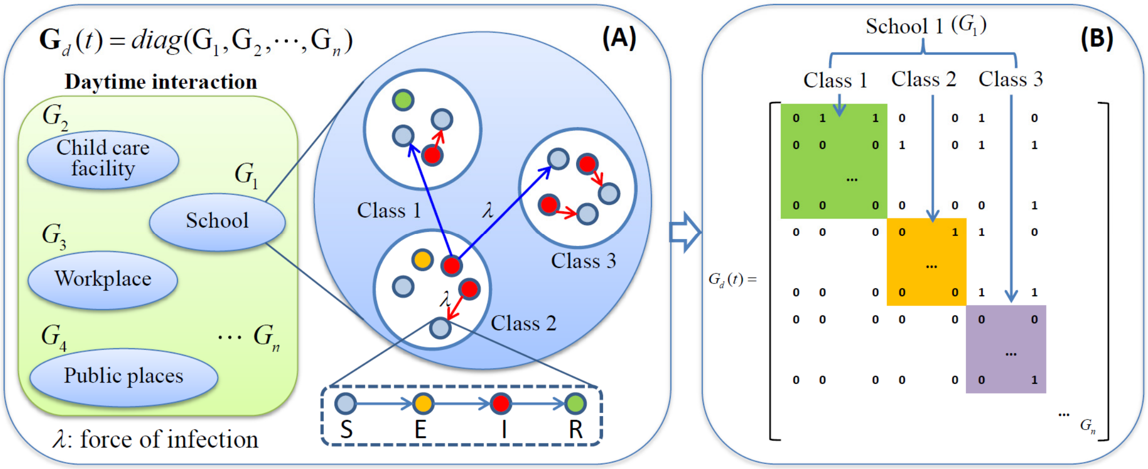

Figure S1. Schematic representation of the social structure describing individuals' daytime activities. (A) A population of individuals is divided into n subpopulations according to the places they go in the daytime. Subpopulations in these places are also divided into several groups. For example, children in school are divided into multiple groups according to the classes they stay in, following a heterogeneous network. Within each group, individuals are identified according to their dynamic status as Susceptible (S), Exposed (E), Infected (I), and Recovered (R). Virus can be transmitted through the interaction between an infected individual and susceptible individuals. (B) The social structure and individuals' interaction is defined in a matrix $\mathbf{G}$, in which the component $g_{ij} = 1$ indicates a connection exists between individual i and j, otherwise $g_{ij} = 0$.

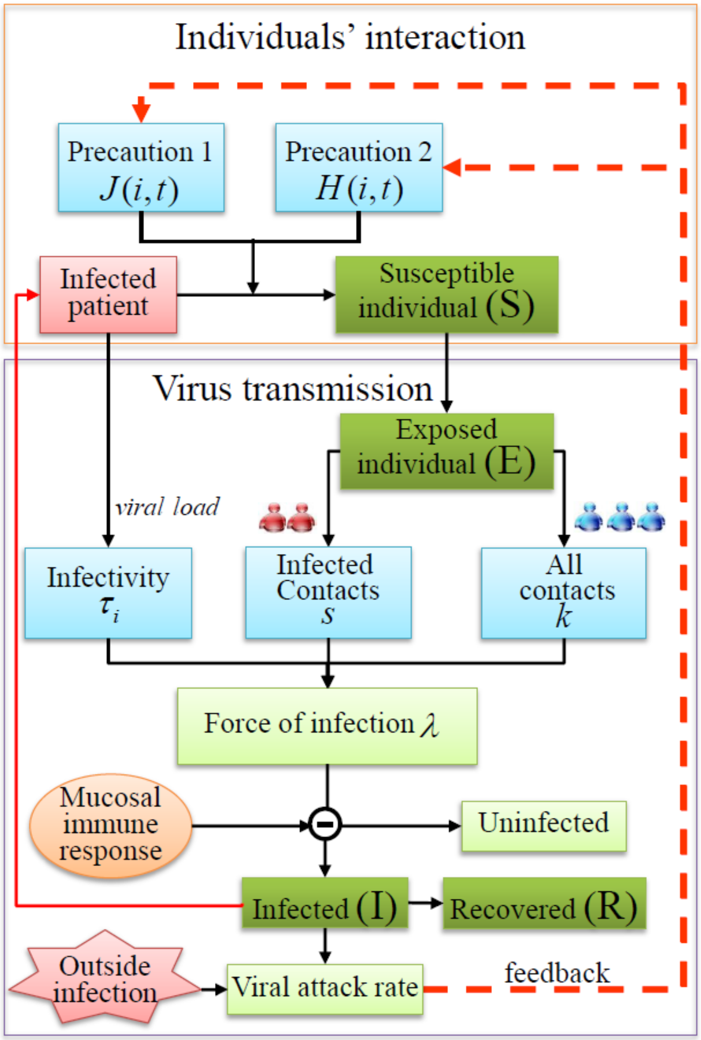

Figure S2. ABM describes individual interaction and virus transmission.

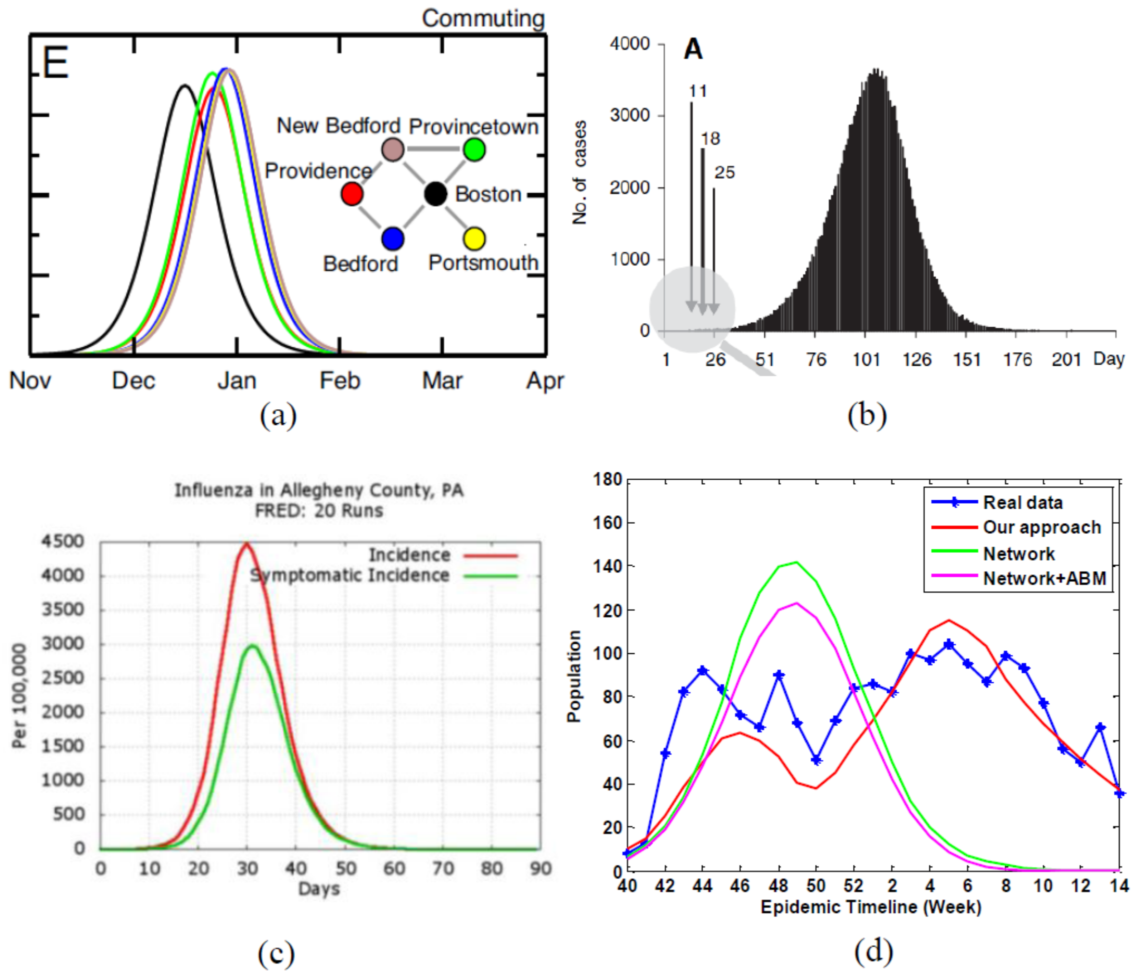

Figure S3. Comparison of current approaches and our HGM approach. (a) The epidemic predictions in six cities using a network modeling method [1]. (b) A simulation of influenza epidemic in a district of a city using census information to build social network [2]. (c) The simulation of an epidemic in a county using synthetized population to build social network and using agent-based model to describe an individual health condition and social behavior [3]. (d) Our approach (red line), network approach (green line), and network+ABM approach (magenta line). From these subfigures we can see that these published methods can only simulate epidemic with one wave, but fails to simulate multiple waves. Their simulations could not reflect the real virus attack rate and present the dynamic change of epidemic. But our simulation result can reflect the dynamic change of epidemics accurately.

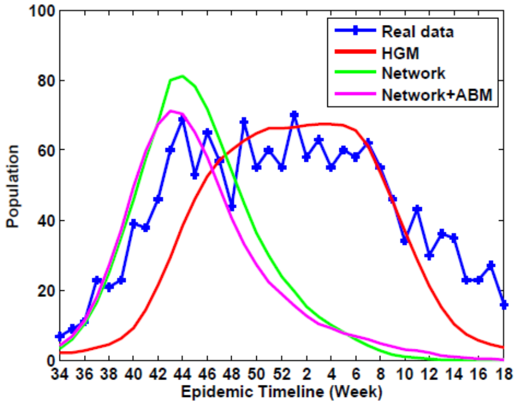

(a)

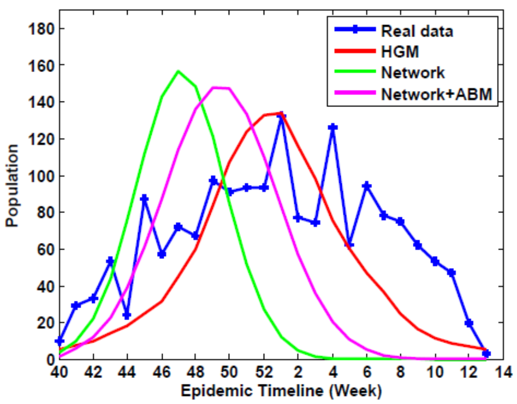

(b)

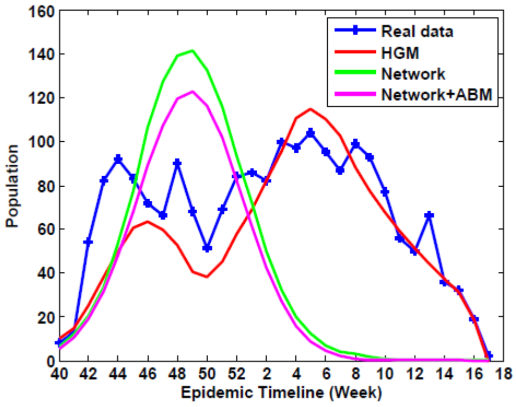

(c)

Figure S4. Comparison of our HGM approach with two common approaches: network based modeling and network combined with an ABM (network+ABM) in the prediction of epidemics in three influenza seasons. (a) 2009-2010 influenza season; (b) 2010-2011 influenza season; and (c) 2011-2012 influenza season. In each subfigure, blue line stands for real clinical data, red line is the predicted epidemic curve using our HGM, green line is the prediction using network based modeling, and magenta line is the prediction using network + ABM. The difference between our HGM and other two methods are (1) we designed an ABM to describe the dynamic process of an epidemic by incorporating factors such as individuals cognition, climate, degree of social network, and daily infection rate into our model. The ABM can create a daily feedback of human behavior to the epidemic. Therefore our method can reflect the dynamic change of an epidemic. (2) Our model was trained and validated by real clinical data, so it is more accurate and robust than common methods.

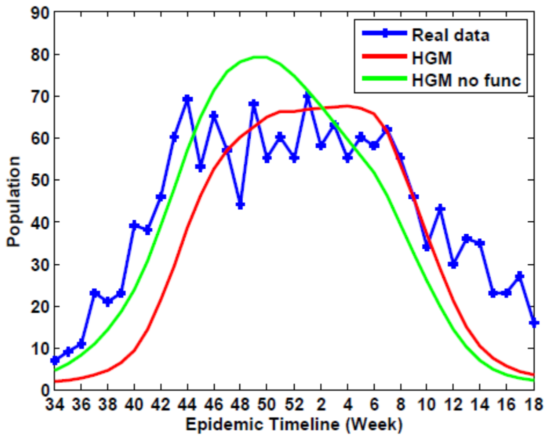

(a)

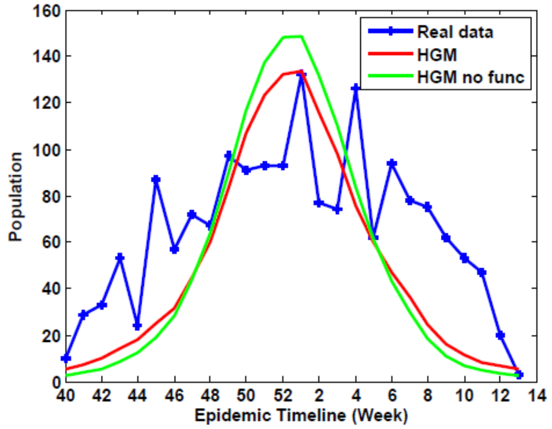

(b)

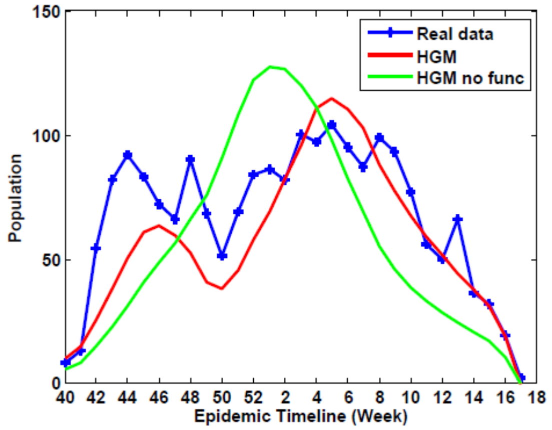

(c)

Figure S5. Measure the function of individual's caution against the disease. (a) 2009-2010 influenza season; (b) 2010-2011 influenza season; and (c) 2011-2012 influenza season. To show our HGM approach can fit the epidemics better than the method without using the function of an individual's caution against the disease, we set the function of an individual's caution against the disease as constant (does not change with daily virus attack rate, climate, and other factors) in our HGM model. We called it *HGM no func*. In each subfigure, green line is the prediction result using *HGM no func* and red line is the prediction result using HGM. It showed that *HGM no func* cannot represent the dynamic change of individuals cognition and caution against the epidemic and failed to models the epidemics with multi-waves, because there is no cognitive and protective behavior preventing the increasing of infections.

Supplementary Tables

Table S1. **Parameters used in the network in three influenza seasons**

	**2009-2010**		**2010-2011**		**2011-2012**	
Contact Probability	p_{in}	p_{out}	p_{in}	p_{out}	p_{in}	p_{out}
Child care center	0.3910	0.0103	0.4010	0.0105	0.4320	0.0108
School	0.1350	0.0305	0.1200	0.0318	0.1410	0.0412
Workplace	0.0637	0.0406	0.0687	0.0421	0.0641	0.0506
Public area on workday	0.0520	0.0520	0.0553	0.0553	0.0524	0.0524
Public area on weekend	0.0729	0.0729	0.0746	0.0746	0.0836	0.0836

Table S2. **Parameters used in the network in three influenza seasons**

	**Proposed value**		
**Symbol**	**2009-2010**	**2010-2011**	**2011-2012**
p_b	0.5	0.5	0.5
σ	0.283	0.283	0.283
α	0.94	0.94	0.94
c_r	6.525	6.329	6.286
c_t	1.3006	1.3004	1.2980
c_{dC}	6.0083	6.0051	6.0150
c_{dH}	6.1602	6.1409	6.1693
c_{dW}	6.8051	6.4333	6.7387
c_a	1.0465	1.1208	1.0728
b_r	-0.1289	-0.1255	-0.1210
b_t	0.5142	0.5440	0.5634
b_d	-0.1015	-0.1025	-0.1021
ε_1	[-0.01~ 0.01]	[-0.01 ~ 0.01]	[-0.01 ~ 0.01]
ε_2	[-0.01 ~ 0.01]	[-0.01 ~ 0.01]	[-0.01 ~ 0.01]

Table S3 **Average connections per person and illness attack rate in five key places and in four age groups in 2010-2011 influenza season**

	Average connections per person			Illness attack rate		
**Places**	**2009-2010**	**2010-2011**	**2011-2012**	**2009-2010**	**2010-2011**	**2011-2012**
Child care center	10.18	11.26	12.19	69.65%	79.39%	79.97%
School	16.49	16.11	17.23	15.83%	14.98%	17.42%
Workplace	4.26	5.01	5.96	8.88%	9.98%	9.36%
Public area on workday	4.94	5.62	6.30	6.61%	8.83%	9.72%
Public area on weekend	7.76	8.01	8.46	7.67%	11.54%	10.73%
Children (0-4)	-	-	-	59.92%	59.58%	63.55%
Children (5-17)	-	-	-	15.86%	14.97%	17.43%
Adults (18-64)	-	-	-	8.83%	9.47%	9.11%
Seniors (65+)	-	-	-	11.28%	11.26%	12.33%

References

1. Balcan D, Colizza V, Gonalves B, et al. Multiscale mobility networks and the spatial spreading of infectious diseases. Proceedings of the National Academy of Sciences. 2009; 106: 21484-21489.
2. Longini IM, Nizam A, Xu S, et al. Containing pandemic inuenza at the source. Science. 2005; 309: 1083-1087.
3. Grefenstette JJ, Brown ST, Rosenfeld R, et al. FRED (A Framework for Reconstructing Epidemic Dynamics): an open-source software system for modeling infectious diseases and control strategies using census-based populations. BMC public health. 2013; 13: 940.
